# Enhancing Checkpoint Inhibitor Therapy in Solid Tissue Cancers: The Role of Diet, the Microbiome & Microbiome-Derived Metabolites

**DOI:** 10.3389/fimmu.2021.624434

**Published:** 2021-07-07

**Authors:** Agnieszka Beata Malczewski, Natkunam Ketheesan, Jermaine I. G. Coward, Severine Navarro

**Affiliations:** ^1^ Icon Cancer Centre, Wesley, Brisbane, QLD, Australia; ^2^ Faculty of Medicine, University of Queensland, Brisbane, QLD, Australia; ^3^ Science and Technology, University of New England, Armidale, NSW, Australia; ^4^ Icon Cancer Centre, South Brisbane, Brisbane, QLD, Australia; ^5^ Department of Immunology, QIMR Berghofer, Brisbane, QLD, Australia; ^6^ Woolworths Centre for Childhood Nutrition Research, Faculty of Health, Queensland University of Technology, South Brisbane, QLD, Australia

**Keywords:** cancer immunotherapy, microbiome, metabolome, checkpoint inhibitor therapy, short chain fatty acids

## Abstract

Host immunity plays a central role in the regulation of anti-tumour responses during checkpoint inhibitor therapy (CIT). The mechanisms involved in long lasting remission remain unclear. Animal studies have revealed that the microbiome influences the host immune response. This is supported by human studies linking a higher microbial richness and diversity with enhanced responses to CIT. This review focuses on the role of diet, the microbiome and the microbiome-derived metabolome in enhancing responses to current CIT in solid tissue cancers. The Western diet has been associated with dysbiosis, inflammation and numerous metabolic disorders. There is preliminary evidence that lifestyle factors including a high fibre diet are associated with improved responses to CIT via a potential effect on the microbiota. The mechanisms through which the microbiota may regulate long-term immunotherapy responses have yet to be determined, although bacterial-metabolites including short chain fatty acids (SCFAs) are recognized to have an impact on T cell differentiation, and may affect T effector/regulatory T cell balance. SCFAs were also shown to enhance the memory potential of activated CD8 T cells. Many therapeutic approaches including dietary manipulation and fecal transplantation are currently being explored in order to enhance immunotherapy responses. The microbiome-derived metabolome may be one means through which bacterial metabolic products can be monitored from the start of treatment and could be used to identify patients at risk of poor immunotherapy responses. The current review will discuss recent advances and bring together literature from related fields in nutrition, oncology and immunology to discuss possible means of modulating immunity to improve responses to current CIT.

## Introduction

Checkpoint inhibitor therapy (CIT) has revolutionized cancer treatment paradigms to date, but much progress remains to be made. In fact, 60-70% of patients do not respond to single agent immunotherapy (1–3). Clinicians are in need of predictive biomarkers in order to successfully identify patients who are most likely to have a long-lasting treatment response (4). Novel therapeutic targets designed to boost responses to existing CIT would enhance and expand therapeutic efficacy and application. Animal studies have confirmed that both spontaneous tumor-specific T cell responses as well as subsequent responses to CIT are microbiota dependent (5, 6). Clinical studies have corroborated these findings with compelling evidence that microbial richness and diversity is associated with a durable response to immunotherapy (7).

Diet remains the major determinant of microbial composition and a high quality diet that is rich in fibre has been associated with improved immunotherapy responses (8). The following review will focus on new developments relating to diet, the microbiome and the microbiome-metabolome with respect to augmenting immunotherapy responses. We will discuss dietary manipulation, use of pre and pro-biotics and fecal transplantation and their potential impact on the outcomes of checkpoint inhibitor therapy. The microbiome-derived metabolome is a new area under investigation and warrants discussion as both a potential novel predictive biomarker and a target for enhancing responses to treatment (9, 10).

## Diet and Responses to Checkpoint Inhibitor Therapy

**Figure 1:**
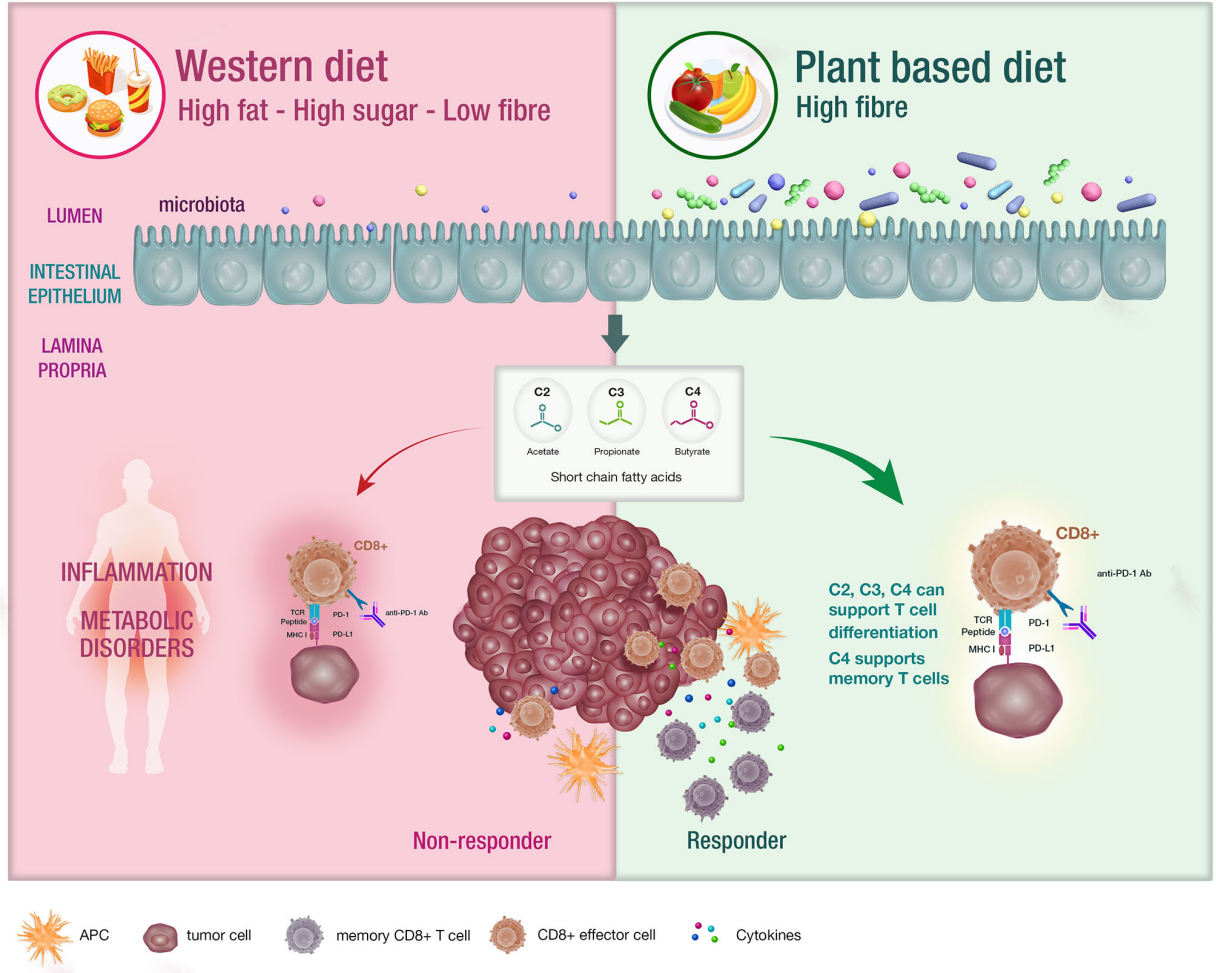
The impact of diet on the microbiota and systemic immunity. Diet has an impact on the host microbiota with the western diet being linked with dysbiosis. Conversely, diets that are high in fibre have been associated with bacterial diversity in the microbiota , which is postulated to support appropriate T cell differentiation. These patients are likely to experience enhanced responses to CIT.

The microbiome is defined as the trillions of bacteria, viruses and fungi colonizing most surfaces of the human body (11). Diet remains the major determinant of the composition of the gut microbiome and a variation in nutrients can induce significant changes within a 24-hour period (12, 13). The standard western diet (WD) which is typically characterized as a high fat, high carbohydrate and low fibre diet. influences the microbiota in many ways including increased bile acid secretion into the gastrointestinal tract, generation of bile-tolerant organisms, dysbiosis and decreased downstream production of short chain fatty acids (SCFA) (14–17) (**Figure 1**).

The intestinal microbiota is a key regulator of immune response during both health and disease (18). For example, the composition of the gut microbiota is known to differ significantly between agrarian and western societies and may provide an explanation for the dramatic rise of allergic and autoimmune disease states in western countries (15, 17, 19–21).

De Filippo et al. (2010) showed that children residing in the remote African community of Burkina Faso (BF) had significantly altered gut microbial composition, compared to those residing in European cities (EU) (19). Key changes for children from BF included enrichment in Bacteroidetes and depletion of Firmicutes (p < 0.001) together with significantly higher SCFA production (p < 0.001) compared to EU children. *Shigella* and *Escherichiae* were also significantly under-represented for BF children. These findings have been attributed to the predominantly plant-based, high-fibre diet that is consumed in rural Africa (19). The health benefits of a high fibre diet have been apparent over many years of research and have been typically associated with increased SCFA production driving regulation of immunological tolerance and promoting gut homeostasis. The impact of diet on checkpoint inhibitor therapy responses is presently under investigation in view of research supporting the importance of the microbiome as a regulator of immune response (8).

Initial data from a study of 113 melanoma patients undergoing CIT confirmed that a high fibre diet was associated with increased microbial richness and highest odds of response (8). Whole grains, fruits and vegetables were associated with a ‘responder’ microbial signature, whilst sugars and processed meat had a negative association (8). Patients who were following a high fibre diet were five times more likely to respond to CIT, compared to patients on a low fibre-diet (OR = 5.3, 95% CI: 1.02 – 26.3) (8).

At present, there are no specific dietary guidelines for patients undergoing immunotherapy and evidence for dietary approaches remains preliminary. Multiple publications over the past 10 years have suggested that dietary fibre and specifically SCFA are key regulators of T cell homeostasis (22–30) (**Table 1**). These early-stage findings from Spencer et al. appear to support the concept that fibre is immunomodulatory (8).

**Table 1:** Summary of studies implicating short chain fatty acids as modulators of T cell differentiation and function.

Study	Summary of study findings
Arpiai et al. (23)	- Butyrate facilitated extrathymic generation of Treg cells - *De novo* generation of Tregs was potentiated by propionate
Furusawa et al. (24)	Butyrate induced the differentiation of Treg cells *in vitro* and *in vivo*
Smith et al. (25)	Short chain fatty acids regulate the size and function of the colonic Treg pool
Park et al. (27)	SCFAs could promote T-cell differentiation into effector or regulatory T cells to promote either immunity or immune tolerance depending on immunological milieu.
Kesphol et al. (29)	- Lower butyrate concentrations facilitated differentiation of Tregs *in vitro* and *in vivo* - Higher concentrations of butyrate promoted IFN-γ-producing Tregs or conventional T cells
Bachem et al. (31)	- SCFA enhance the memory potential of antigen-activated CD8^+^ T cells - Butyrate promoted memory potential of activated CD8^+^ T cells, enhanced metabolism and promoted long-term survival as memory cells.

## Impact of Short Chain Fatty Acids on Immunity and T Cell Function

SCFA including acetate, butyrate and propionate are the bacterial metabolites of fermented fibre and are known regulators of T cell differentiation and function (22–31) (**Figure 1**). Exactly how a high fibre diet enhances checkpoint inhibitor therapy, which relies on CD8+ T cell infiltration into the tumor requires further investigation. Pre-clinical studies suggest that the effects of SCFA on T cell differentiation may depend on the immunological context and the concentration of the relevant SCFA (27, 29). Park et al. (27) showed that T cell differentiation is affected by the local cytokine milieu and that SCFA could promote either effector or regulatory T (Treg) cell development. Kesphol et al. (29) showed that the effects of butyrate on T cell differentiation were in fact dose dependent. Physiological concentrations of butyrate could promote Treg production, whereas higher butyrate concentrations could enhance development of IFNγ producing Tregs or conventional T cells. SCFA have also been shown to enhance generation of macrophage and dendritic cell precursors *via* systemic effects on bone marrow haemataopoiesis (30). Most recently, Bachem et al. (31) showed that the microbiota could affect CD8+ T cell function. In particular, butyrate was shown to enhance the memory potential of activated CD8+ T cells (31).

Enhancing immunotherapy responses through use of dietary fibre and its impact on SCFA production requires further study, although this would be a straight forward and risk averse intervention. Elucidating the mechanisms through which fibre may enhance treatment responses is likely to be a more complex question that may be addressed further through study of the microbiome-derived metabolome in humans (9, 10).

## Antibiotics Impair Immunotherapy Outcomes

Dysbiosis and perturbation of gut flora is a known consequence of antibiotic use (32). Infection and antibiotic use are common occurrences during the course of cancer treatment. There is now a well-documented association between antibiotic use and poor therapeutic outcome. Patients with antibiotic exposure have impaired treatment responses, including decreased response rates, shorter progression free and diminished overall survival (33–38). There is a wide variation in the cited overall survival times for solid tissue cancers treated with CIT for groups with and without antibiotic exposure (34). Nonetheless, the impact of antibiotics appears to be clinically significant and detrimental. In a large, retrospective study of 568 stage III and stage IV melanoma patients, the authors confirmed that the antibiotic exposed group had a significantly worse OS of 27.4 months, compared to 43.7 months for the antibiotic-unexposed group (hazard ratio 1.81, 95% confidence interval 1.27-2.57, *p* < 0.001) (38). At this stage, mechanistic studies are lacking and most of the available data is retrospective (33). There is also a possibility that patients requiring antibiotic therapy are potentially a more unwell group in terms of susceptibility to infection and compromised immunity leading to suboptimal treatment outcomes (33). Further clinical trials are awaited to determine which interventions could be implemented to improve clinical outcomes for patients who find themselves in this common scenario.

## The Microbiome and Regulation of Responses to Checkpoint Inhibitor Therapy

In 2015, landmark pre-clinical mouse studies confirmed that the anti-tumor effects of CTLA-4 and PD-L1 blockade were facilitated by commensal intestinal flora (5, 6). Sivan et al. (2015) studied two groups of genetically similar mice from two different commercial sources (The Jackson Laboratory (JAX) and Taconic Biosciences (TAC)) with distinct microbiota composition (5). At baseline, JAX mice and TAC mice were noted to have significantly different rates of spontaneous melanoma growth. This was attributed to differences in spontaneous immunity between the two groups. High intra-tumoral CD8 T cell infiltration was associated with low melanoma growth (JAX mice), whilst low intra-tumoral CD8 T cell infiltration was associated with accelerated melanoma growth (TAC mice) (5). These differences in T cell immunity were shaped by the composition of the microbiota. Fecal microbiota transplant (FMT) of JAX mice (via oral gavage) to TAC recipients was sufficient to augment CD8+ T cell infiltration into tumor, and slow the melanoma growth rate to the same extent as treatment with an anti-programmed-death-ligand-1 antibody (anti-PD-1 Ab). *Bifodobacterium* were identified as being critical to antitumor immunity and could mediate therapeutic effects by enhancing host antitumor T cell responses including peripheral T cell induction, CD 8+ T cell infiltration into tumor and dendritic cell activation, which led to enhanced CD8+ T cell priming. Interestingly, Vetizou et al. (2015) (6) found that responses to CTLA-4 blockade were dependent on the presence of *Bacteroides*. In this study, therapeutic responses to anti-CTLA-4 mAb were tested in germ free and antibiotic treated mice. The anti-tumor effects of anti-CTLA-4 mAb were significantly compromised in these two groups but could be re-established following colonization with *B. fragilis* (6).

Since 2015, multiple clinical studies have supported the findings that the efficacy of CIT is microbiota dependent (7, 39–42). Whilst a single, consistent microbial ‘responder’ signature has not been identified, microbial richness with a high alpha diversity has been key findings associated with CIT responsiveness (7). Alpha diversity refers to the ecological richness of a given microbiome sample (43) as opposed to beta diversity, which is the diversity of microbes between two different samples. Bacterial species that have been associated with a treatment response have included taxa within the Ruminococaceae family of the Firmicutes phylum (44). A lack of response has been associated with bacterial taxa within the Bacteroidales order of the Bacteroidetes phylum (44). Investigators have noted that there is an absence of definitive overlap between responder microbial signatures as described in various clinical studies, suggesting that efficacy may not rely entirely on a specific strain of bacteria but more likely on how the microbiome interacts with the immune system *via* the production of metabolites. It is likely that a favorable microbiome can lead to enhanced antigen presentation and effector T cell function leading to improved local anti-tumor responses and systemic immunity (7). Historically, tumor infiltration with CD8+ T cells has been associated with a favorable prognosis, which is in agreement with recent findings in immunotherapy (45–48).

The microbiota is an exciting therapeutic target that could be of enormous value. Mouse studies have demonstrated the role of FMT as a means of altering the microbiota to successfully effect tumor control. Whilst FMT is an established technique for treatment of refractory *C Difficile* infection (CDI), it is not a standard technique for other dysbiotic states (49). Further study is needed to investigate the mechanisms through which FMT is able to reconstitute a functional gut microbiome in the CDI setting. There may be unique ecological factors during CDI that render FMT effective, whereas in other dysbiotic states, such as ulcerative colitis, results have not been as great. Other more practical issues with FMT will include presence or absence of facilities to carry out the procedure, our lack of understanding of what constitutes an ideal donor and the obvious difficulties with standardization of fecal donor specimens (50). Clinical trials (NCT03353402; NCT03341143) are presently underway using fecal donor material from complete responders and a phase I trial has been completed (NCT03353402) confirming safety of this procedure in a small group of patients (51). We suggest that the fecal and serum microbiome-derived metabolome will provide greater insight into the functional metabolic products of specific microbial communities and will be able to quantify these, providing researchers with new therapeutic applications.

Probiotics are defined as live organisms that are taken orally in order to provide health benefits to the host. Conventional probiotics are available over the counter and usually contain limited bacterial strains. Probiotics have been associated with detrimental effects during checkpoint inhibitor therapy including lower microbiome diversity (8). Whilst murine models have confirmed the proof of principle in that certain commensals appear essential to immunotherapy responses, the complexity of the microbiome in humans is such that it would be unrealistic to re-create a responder phenotype with conventional probiotics. In an elegant study, Suez et al. (2018) randomized healthy human volunteers to treatment with broad spectrum antibiotics followed by either watchful waiting, FMT or treatment with an 11-strain probiotic cocktail (52). FMT resulted in rapid reconstitution of indigenous microbial flora, whereas probiotics resulted in significant delays to reconstitution of normal flora that lasted up to 5 months post probiotic cessation. At present, patients on CIT should be cautioned against the use of probiotics as they appear to be detrimental in this setting.

## The Microbiome-Derived-Metabolome

The metabolome is a relatively new concept that describes the metabolites in a biological system. Metabolomics is performed utilizing mass spectrometry based techniques and can look at the end metabolic products of gut bacteria (either fecal or serum samples). These may represent the metabolic end products of the bacteria that are present. As the ideal ‘microbial’ responder signature has not been identified, there is a possibility that different bacterial communities may ultimately exert similar immunologic outcomes through common metabolic end products, such as SCFA.

Investigators have assessed the role of the microbiome-derived metabolome in patients undergoing anti-PD-1 therapy and have found that those who were classed as good responders had higher levels of SCFA, compared to patients who had early progressive disease (9, 10). Nomura et al. (2020) assessed serum and fecal metabolites in 52 patients with mixed solid tissue cancers undergoing single agent immunotherapy (9). Fecal concentrations of acetic acid, propionic acid, butyric acid, valeric acid (*p* =0.05 - 0.002) and plasma isovaleric acid (*p* < 0.01) were associated with significantly prolonged progression free survival times (9). Botticelli et al. (2020) showed that non-small cell lung cancer (NSCLC) patients (n = 11) who had early disease progression within 3 months of starting nivolumab had fecal samples that were characterized by low levels of SCFAs (propionic, butyric, acetic, valeric acids), compared to long-term responders (progression free survival > 12 months) (10). In contrast to these studies, a separate group, which looked at (mostly melanoma) patients undergoing anti-CTLA-4 Ab monotherapy (n = 85) found that elevated levels of SCFAs were associated with disease progression (53). In this study, low baseline butyrate and propionate were associated with longer PFS (*p* = 0.0015 and *p* = 0.0029 respectively). Much larger studies with a single-tumor focus looking at both single agent and combination immunotherapy are required to confirm these findings.

We postulate that the microbiome-derived-metabolome is a predictive biomarker of response and may be able to identify patients who are at greater need of early intervention (e.g. dietary) in order to augment immunotherapy responses. Serial monitoring of the microbiome-metabolome may also be possible during a patient’s treatment in order to assess levels of SCFA as a guide to immunological response.

A therapeutic application is a theoretical possibility. Metabolites can be more readily quantified and regulated compared to complex bacterial ecosystems and may be easier to manipulate in order to induce an immune response. SCFA administration has been utilized in the setting of dysbiosis with autoimmune bowel disease (54). Whilst results have not been favorable to date, the exploration of this approach has not been complete (54).

## Discussion

The advent of CIT has led to a new exploration of the host-tumor relationship and has raised many questions over what drives an effective host immune response. We now know, perhaps unsurprisingly, that both animals and humans with better baseline systemic and anti-tumor immunity go on to have better responses to CIT.

A key question is how to identify immunotherapy responders and more importantly, how to improve clinical outcomes for the non-responders. We have a range of different targets that could be manipulated although we are still awaiting the results of multiple studies that will direct our approach.

The function of the immune system is inextricably linked to the microbiota and we have clear evidence that perturbation of bacterial ecology through antibiotics has functional implications for immunotherapy, whereas potential enhancements could be achieved through nutritional manipulation.

Clinicians and scientists continue to search for a consistent, responder microbiome signature, although it is likely that there is more than one microbial profile that may be associated with good anti-tumor immunity. Microbial communities ultimately exert their effects through metabolic end products such as SCFA and different congregates of micro-organisms may produce the same beneficial metabolites.

Preliminary findings suggest that fibre is emerging as a modulator of immune response and this may not be surprising given the plethora of health benefits that have been associated with a plant-based, high-fibre diet. Further mechanistic studies are needed to define the immunomodulatory role of fibre, given the fact that it has traditionally been associated with Treg induction and immunological tolerance. Recent mouse studies have confirmed that SCFA including butyrate have activity in enhancing CD8+ T cell memory function and this has helped to shed light on the complex role that SCFA have in immune regulation (31).

FMT is presently under study as a technique for enhancing tumor control by introducing a responder microbiome. It is certainly appealing as a means of replacing an entire microbiota with one from a known immunotherapy-responder. Whilst this technique been successful in the setting of refractory CDI, it is unclear whether this FMT will gain traction in the immunotherapy setting, given its inconsistent results in other dysbiotic states.

The microbiome-derived-metabolome is a new concept that refers to measurement of the metabolic end products of the microbiome in either serum or feces. Metabolites, such as SCFA can be quantified and may have a potential application as a predictive biomarker and as a target for manipulating the host immune response. Furthermore, the adaptive mature of the microbiome-immune cross talk suggests that metabolite-based therapeutics might offer attractive new therapeutic avenues to enhance the immune response to CIT and provide a positive feedback signal to the microbial ecosystem, possibly extending the duration of therapeutic benefit.

The future may hold baseline microbiome and microbiome-metabolome profiling of patients at baseline as well as at several time points throughout their immunotherapy treatment. Correlation of the metagenomic and metabolomic aspects of the microbiome is required in order to have a better functional understanding of the human immune response during CIT.

## Author Contributions

AM and SN prepared the manuscript. NK and JC provided intellectual and editorial feedback. All authors contributed to the article and approved the submitted version.

## Funding

SN is supported by a Fellowship from the Children’s Hospital Foundation (RCP10317).

## Conflict of Interest

SN is funded by the Queensland Children's Hospital Foundation through philanthropic funding from Woolworths. The CHF and Woolworths were not involved in any stage of this research.

The remaining authors declare that the research was conducted in the absence of any commercial or financial relationships that could be construed as a potential conflict of interest.
